# Recipient microbiome-related features predicting metabolic improvement following fecal microbiota transplantation in adults with severe obesity and metabolic syndrome: a secondary analysis of a phase 2 clinical trial

**DOI:** 10.1080/19490976.2024.2345134

**Published:** 2024-04-29

**Authors:** Zhengxiao Zhang, Valentin Mocanu, Edward C. Deehan, Naomi Hotte, Yuanyuan Zhu, Shanshan Wei, Dina H. Kao, Shahzeer Karmali, Daniel W. Birch, Jens Walter, Karen L. Madsen

**Affiliations:** a College of Ocean Food and Biological Engineering, Fujian Provincial Engineering Technology Research Center of Marine Functional Food, Jimei University, Xiamen, Fujian, China; b Department of Medicine, University of Alberta, Edmonton, AB, Canada; c Department of Surgery, University of Alberta, Edmonton, AB, Canada; d Department of Food Science and Technology, University of Nebraska, Lincoln, NE, USA; e Division of Gastroenterology, Department of Medicine, University of Alberta, Edmonton, AB, Canada; f APC Microbiome Ireland, School of Microbiology and Department of Medicine, University College Cork, Cork, Ireland

**Keywords:** Fecal microbiota transplantation, gut microbiota, dietary fiber, metabolic syndrome, obesity

## Abstract

Microbial-based therapeutics in clinical practice are of considerable interest, and a recent study demonstrated fecal microbial transplantation (FMT) followed by dietary fiber supplements improved glucose homeostasis. Previous evidence suggests that donor and recipient compatibility and FMT protocol are key determinants, but little is known about the involvement of specific recipient factors. Using data from our recent randomized placebo-control phase 2 clinical trial in adults with obesity and metabolic syndrome, we grouped participants that received FMT from one of 4 donors with either fiber supplement into HOMA-IR responders (*n* = 21) and HOMA-IR non-responders (*n* = 8). We further assessed plasma bile acids using targeted metabolomics and performed subgroup analyzes to evaluate the effects of recipient parameters and gastrointestinal factors on microbiota engraftment and homeostatic model assessment of insulin resistance (HOMA2-IR) response. The baseline fecal microbiota composition at genus level of recipients could predict the improvements in HOMA2-IR at week 6 (ROC-AUC = 0.70). *Prevotella* was identified as an important predictor, with responders having significantly lower relative abundance than non-responders (*p* = .02). In addition, recipients displayed a highly individualized degree of microbial engraftment from donors. Compared to the non-responders, the responders had significantly increased bacterial richness (Chao1) after FMT and a more consistent engraftment of donor-specific bacteria ASVs (amplicon sequence variants) such as *Faecalibacillus intestinalis* (ASV44), *Roseburia* spp. (ASV103), and *Christensenellaceae* spp. (ASV140) (*p* < .05). Microbiota engraftment was strongly associated with recipients’ factors at baseline including initial gut microbial diversity, fiber and nutrient intakes, inflammatory markers, and bile acid derivative levels. This study identified that responders to FMT therapy had a higher engraftment rate in the transplantation of specific donor-specific microbes, which were strongly correlated with insulin sensitivity improvements. Further, the recipient baseline gut microbiota and related factors were identified as the determinants for responsiveness to FMT and fiber supplementation. The findings provide a basis for the development of precision microbial therapeutics for the treatment of metabolic syndrome.

## Introduction

Obesity is a complex chronic progressive condition characterized by excess adiposity and dysregulation in enteroendocrine and neurohormonal signaling pathways favoring increased appetite and energy storage.^1,2^ There exists substantial evidence that the gut microbiota affects all aspects of host energy homeostasis through diverse mechanisms involving effects on immune, hormonal, and neural systems that effect adipose tissue, muscle, and liver.^3^ Generally, studies show individuals with obesity to have decreased bacterial diversity and gene richness along with functional microbial metabolic alterations and specific changes in microbial profiles that associate with host metabolic dysregulation.^4–8^ A recent systematic review concluded that Proteobacteria was the most frequently associated phylum in subjects with obesity, likely related to the commonly observed chronic proinflammatory state seen in subjects with obesity. Although evidence from animal models has described potential causative relationships between altered gut microbiota, obesity, and metabolic syndrome,^3,9^ convincing causal relationships remain to be fully elucidated in human clinical trials.^10–12^

Fecal microbiota transplantation (FMT) is a microbial-based strategy that aims to restore a disrupted gut microbial ecosystem through the transfer of whole, non-defined stool.^13^ FMT has proven to be highly effective in recurrent *Clostridioides difficile* infection (rCDI), with a success rate of approximately 80–90% after a single treatment.^14^ A recent meta-analysis of randomized placebo-controlled trials using FMT to treat obesity and metabolic syndrome concluded that FMT can induce short-term improvements in insulin sensitivity and glucose homeostasis in patients with metabolic syndrome and obesity.^15^ However, responses to FMT remain variable with some subjects responding favorably and others showing no response.^16,17^ In addition, it is still not well understood why the observed short-term benefits of FMT on dysglycemia do not persist over time. One possibility is that the lifestyle of the host including diet causes a reversion of changes induced by FMT. Other emerging work suggests that pre-FMT recipient microbiota composition^18^ and donor-recipient complementarity^19,20^ may be key determinants of FMT success. However, gut microbial composition and functions are strongly influenced by host inflammation, diet habits, and other environmental exposures.^21^ Thus, it is important to address interactions between recipient factors and microbial engraftment and how these features associate with FMT-induced metabolic benefits in patients with obesity and metabolic syndrome in order to fully maximize these types of therapies.

We recently carried out a proof-of-principle clinical trial showing that low-fermentable fiber (cellulose) was effective in the induction and maintenance of FMT-induced metabolic improvement in patients with obesity and metabolic syndrome.^22^ In order to address interactions between recipient factors and microbial engraftment which were associated with metabolic benefits, we conducted a secondary analysis by stratifying FMT recipients based on their response to the FMT intervention as either an improvement (responders) in the homeostatic model assessment of insulin resistance (HOMA2-IR) or no response (non-responders). Here, we compared the extent of the microbial engraftment after FMT between responders and non-responders and then evaluated the effects of the recipients’ baseline parameters on clinical responses.

## Results

**Figure 1.**
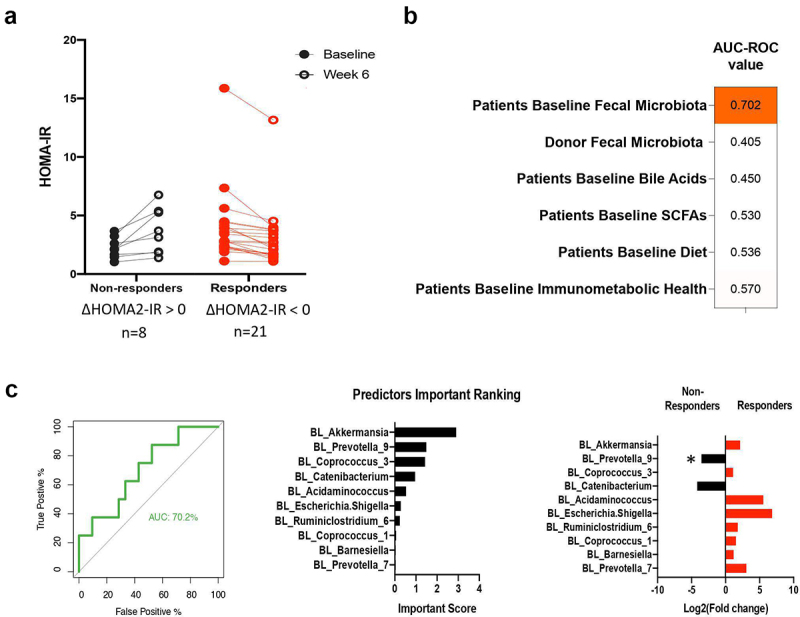
Identification of FMT recipient factors that predict HOMA-IR responses by machine learning.

A double-blind, randomized, placebo-controlled clinical trial using FMT and fiber supplementation was conducted between 2018 and 2019 in participants (age 47.8 ± 10.0 years, female sex predominance 83.6%) with severe obesity (BMI 45.3 ± 7.0 kg/m^2^) and metabolic syndrome recruited from Bariatric Clinic in Edmonton, Alberta, Canada. Patients were given a single dose of FMT (50 g of donor stool) with 20 capsules orally from one of 4 donors, followed by 6 weeks of daily fiber supplementation with either a fermentable fiber mixture (soluble corn fiber, resistant starch type 4, and acacia gum) or a low fermentable fiber (microcrystalline cellulose).^22^ In the current analyzes, participants receiving FMT were divided into responders and non-responders based on their HOMA2-IR response (Figure 1a). HOMA2-IR and insulin baseline values were different between the two groups and subjects were on various medications including metformin, GLP-1 agonists, and SGLT2 inhibitors.^22^ There was no difference between the groups in the number of subjects on each of these types of medications and there was no influence of medication on response as analyzed using multivariate logistic regression.^22^ No other baseline characteristics showed significant differences (Table S1). Fecal microbiota composition using 16S rRNA gene amplicon sequencing, and biomarkers of host–microbiota interactions, which were suggested to contribute to the pathophysiology of obesity, were integrated in this study.

Line graphs show differences in the effects of (a) FMT with fiber supplementation on HOMA-IR for responders and non-responders, as defined according to changes in HOMA-IR from baseline to week 6 (decreased vs. increased). AUC-ROC values show the performance accuracy of random forest classifiers for predicting responders from non-responders in (b) FMT with fiber supplementation induced HOMA-IR attenuation. Prediction performance of random forest classifiers trained to predict FMT plus fiber responders in HOMA-IR. (c) (left) AUC-ROC curves show the performance accuracy of random forest classifiers trained to predict responders vs. non-responders for HOMA-IR with FMT plus fiber treatment using the relative abundance of the patient’s baseline fecal microbiota at genus level; (center) Horizontal bars represent the top 10 important taxa for predicting responses; (right) Horizontal bars represent taxa comparisons between responders and non-responders. * Indicates *p* value < .05 using Wilcoxon test. Δ, absolute change from baseline to week 6; AUC-ROC, area under the receiver operating characteristic curve; HOMA-IR, homeostatic model assessment of insulin resistance; FMT, Fecal microbiota transplant.

### Identification of microbiota-related baseline predictors of HOMA2-IR response

To gain insight into the involvement of the baseline recipient and donor gut microbiota in the physiological effects of FMT, a machine learning approach was employed to identify predictors for the individual HOMA2-IR response. The models included variables pertaining to microbiota composition (for both donor and recipient fecal microbiota), functional features (fecal SCFAs and bile acids), immunometabolic health markers (inflammation, lipids, metabolic markers), and dietary nutrient and food group intake data.

Random forest classifications trained on the recipient’s baseline microbiota composition were the most predictive of HOMA2-IR response (Figure 1b, ROC-AUC = 0.70). Regarding the impact of FMT with fiber on HOMA2-IR, the ten bacterial taxa with the highest predictive value at the genus level were determined (Figure 1c). At baseline, the relative abundance of *Prevotella_9* was significantly lower in responders (*p* = 0.017). In contrast to microbial composition, microbial functional features (SCFA and bile acids), immunometabolic markers and macronutrient consumption data of participants did not predict their response to FMT with fiber supplementation (ROC-AUC <0.6). Together, our findings indicate that the recipient microbiota at baseline had a relatively high degree of impact on the insulin-sensitizing effects of FMT.

### Microbial diversity changes in FMT recipients associated with HOMA-IR response

**Figure 2.**
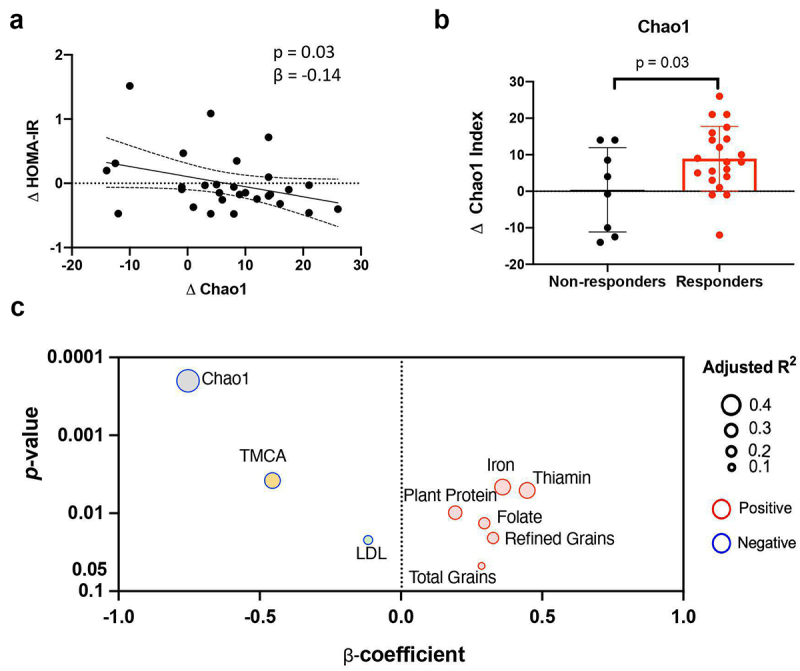
FMT recipient factors at baseline are associated with the differences in fecal microbiota changes in diversity between responders and non-responders in HOMA-IR.

FMT is an untargeted strategy aimed at manipulating gut microbial ecology on a wider scale. Therefore, we investigated the association between the change in α-diversity (Chao1 index) and the response in HOMA2-IR following FMT. After receiving FMT, responders demonstrated a decrease in HOMA2-IR while their fecal microbial diversity increased (*p* = .03, repeated measures linear mixed model regression correcting for donor selection, Figure 2a). Moreover, within the FMT group, we observed that the HOMA2-IR responders had substantially greater increases in microbial richness than the non-responders (*p* = .03, Figure 2b).

(a) Microbial diversity is associated with improvement in HOMA-IR in patients receiving FMT with fiber supplementation. Analyzed using repeated measures linear mixed model regression after adjusting for donor selection. (b) Comparison of shifts in fecal alpha-diversity (Δ week 6 - baseline) between responders and non-responders, as defined according to changes in HOMA-IR from baseline to week 6 (decreased vs. increased). Statistical comparisons between groups were by ANCOVA adjusted for donor selection. (c) Scatter plots show recipient parameters that predict the changes in microbial diversity after FMT using baseline intakes of calorie-adjusted macronutrients and baseline clinical measurements including anthropometric measurements, immune cytokines, metabolic makers, and fecal bile acids (15 compounds), as well as biomarkers of host-microbiota interactions. Analyzed using repeated measures linear mixed model regression after adjusting for donor selection. LDL, low-density lipoprotein cholesterol; TMCA, tauro-muricholic acid.

### Recipient factors influencing microbiota diversity increase after FMT

We next assessed whether baseline host parameters such as diet, immunological status, bile acid profiles, and gut microbiota were correlated with the α-diversity response to the 6-week FMT intervention. The microbial diversity at the pre-FMT baseline was the strongest factor inversely associated with the diversity changes following the FMT (*p* = .0002, r_s_ = −0.75, Figure 2c). In addition, levels of tauro-muricholic acid (TMCA) at baseline were inversely correlated with the α-diversity improvement (*p* = .004, r_s_ = −0.46). In contrast, an increase in α-diversity was positively associated with baseline dietary components such as plant protein, total grains, refined grains, and numerous mineral elements (iron, thiamin, and folate; *p* < .05). Thus, the degree of change in diversity was highly dependent on the baseline status of microbial diversity, diet, and lipid metabolism in the recipients prior to FMT.

### Bacterial engraftment success and changes are associated with HOMA2-IR response

**Figure 3.**
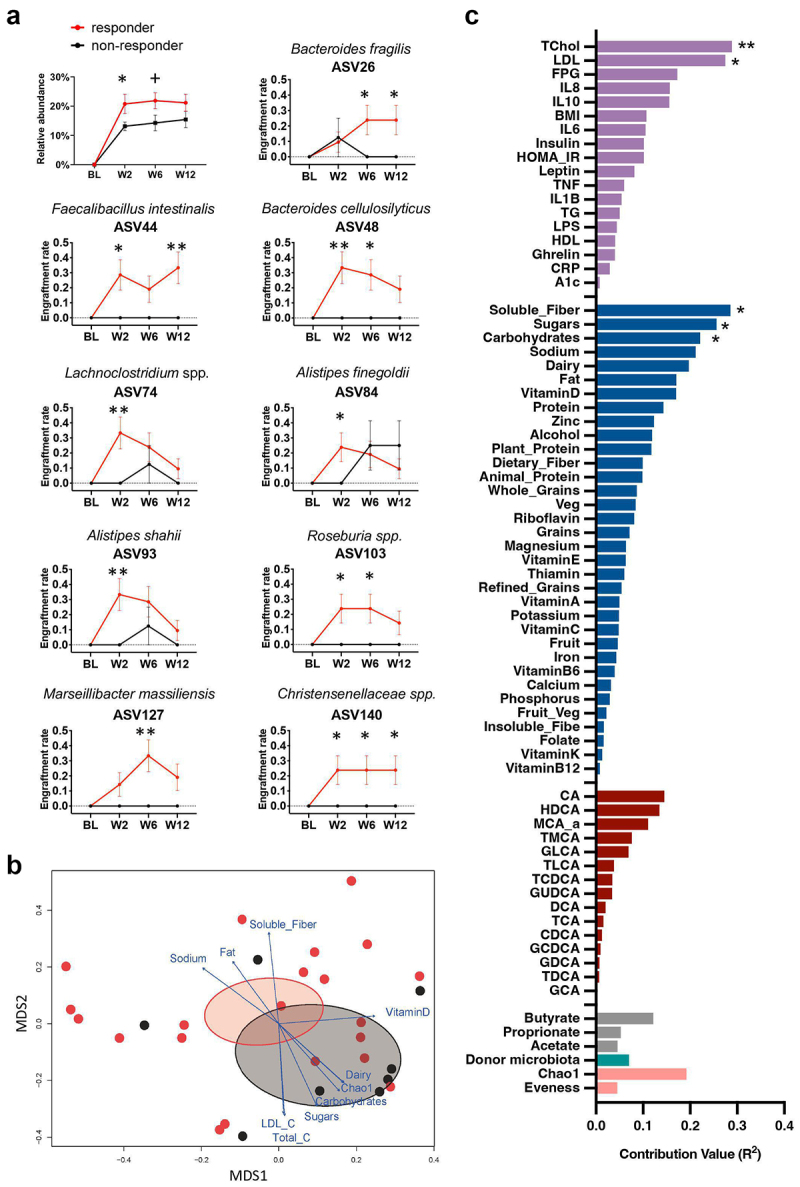
FMT recipient baseline factors predict donor-specific ASVs engraftment.

To determine if microbiota engraftment was associated with HOMA2-IR improvement in obesity and metabolic syndrome, we first evaluated the dynamics of bacterial Amplicon sequence variants (ASVs) derived from patient and donor after FMT with fiber supplementation. Second, we assessed if the extent of bacterial ASVs shifts following FMT was associated with individual HOMA2-IR responses. During the 12 weeks, changes in the fecal microbiota of recipients following FMT were evaluated using pre-FMT samples from each donor-recipient pair as a baseline. The results revealed that microbial engraftment at ASV levels differed across recipients, which was mostly represented in variable proportions of donor-specific ASVs (species shared only with the donor) with a considerable fluctuation over the study period of 12 weeks (Figure S1). Based on the clinical response, responders demonstrated greater increases in the proportion of donor-specific ASVs colonization than non-responders at weeks 2 and 6 (Figure 3a, *p* = .044 and *p* = .057). These changes in microbiota persisted for six weeks following treatment.

(a) Comparison of the donor-specific ASVs fraction retained in the FMT-recipients between responders and non-responders over the 12 weeks. Line charts compare the ratio of FMT recipients who retained donor-specific ASVs in total number of the recipients (engraftment rate) among responders vs. non-responders. ASVs that had a significantly higher engraftment rate in the responders relative to the non-responders were selected (*p* < .05). Statistical analysis was performed by repeated measures ANOVA models for assessing the main factor effect. Comparisons were made between the two groups at each time point by *t*-test with Welch correction. Data are reported as mean ± SEM [+*p* < .1, *p < .05, **p < .01]. ASV26: *Bacteroides fragilis*; ASV44: *Faecalibacillus intestinalis*; ASV48: *Bacteroides cellulosilyticus*; ASV74: *Lachnoclostridium* spp.; ASV84: *Alistipes finegoldii*; ASV93: *Alistipes shahii*; ASV103: *Roseburia* spp.; ASV127: *Marseillibacter massiliensis*; ASV140: *Christensenellaceae* spp. ASV, amplicon sequence variant. (b) Weighted metric multidimensional scaling based on Bray-Curtis dissimilarity and environmental fitting test analysis (envfit) displaying top 10 recipient baseline factors explaining donor-specific ASVs distribution variation between responders and non-responders at week 6. (c) The figure C evaluated contribution degrees of baseline factors affecting the donor-specific ASVs community in the FMT recipients (measured using coefficient of determination (R2)). And the significant association was tested using permutation test (*n* = 999) in the vegan package [*p < .05, **p < .01].

Several microbial species engraftments differed significantly between responders and non-responders over the 12 weeks, including *Faecalibacillus intestinalis* (ASV44) having the glycosyl-transferring function,^23^ the butyrate-producing taxon *Roseburia* spp.^24^ (ASV103), and *Christensenellaceae* spp., which are negatively associated with obesity^25^ (ASV140) (Figure 3a, *p* < .05). These donor-specific ASVs increased significantly in the responders but not in the non-responders, indicating that the donor bacteria were able to colonize and coexist with recipient microbiota in the responders. Overall, the durability of donor-specific ASVs engraftment in the feces of the recipients varied widely.

In addition, we determined which bacteria of the recipients were removed after the FMT with fiber intervention. Figure S2a demonstrates the variations in the initial number of fecal ASVs among recipients during the intervention, with no obvious distinction between responders and non-responders (Figure S2b). Nevertheless, upon conducting a comparison of individual ASVs, we noted that after six weeks of treatment, the non-responders exhibited significant reductions in the presence of three ASVs, including a fat-utilizing species *Alistipes finegoldii*^26^ (ASV84), and two fiber-degrading species *Ruminococcus callidus*^27^ (ASV143) and *Bacteroides intestinalis*^28^ (ASV148) (Figure S2B). The significant removal of these ASVs from non-responders but not from responders suggests that these ASVs may have a beneficial effect on the metabolic status of the patient and that their disappearance may be associated with the lack of insulin sensitivity improvement observed in non-responders.

### Recipient factors influencing bacterial ASVs engraftment after FMT

While engraftments in specific bacteria changes were associated with improvements in insulin sensitivity, the modification of the microbiome by FMT plus fiber supplementation was not maintained following cessation of fiber intake in our cohort. We evaluated the potential influence of several baseline host variables, such as immune function, lipid metabolism, plasma bile acids, diet, and microbial diversity, on the presence of donor-specific ASVs following FMT.

We first applied weighted metric multidimensional scaling (MDS) ordination to the Bray-Curtis dissimilarity matrix of overall the engrafted donors ASVs community between FMT patients at the week 6. Then, vectors were projected into this ordination space to represent the correlation between baseline factors and donor-specific ASV community variation of responders and non-responders (Figure 3b). This environmental fitting test analysis (envfit function in Vegan package) revealed that baseline dyslipidemia-related markers total cholesterol (R^2^ = 0.29 and *p* = .009) and LDL (R^2^ = 0.27 and *p* = .016), and dietary intakes of soluble fiber (R^2^ = 0.29 and *p* = .014), sugars (R^2^ = 0.26 and *p* = .012) and carbohydrates (R^2^ = 0.22 and *p* = .029) were key baseline factors predicting the donor-specific ASVs community variation (*p* < .05; Figure 3c). According to the vector direction of these factors, the donor ASVs engrafted community in responders is strongly associated with high soluble fiber intake, whereas high dyslipidemia markers and high intake of sugars and carbohydrates contribute more to the non-responder’s unsuccessful donor ASVs engraftment status.

We then utilized a repeated measures linear mixed model regression following controlling for donor selection with FDR correction to assess the association of baseline factors with each specific donor ASVs changes (Figure S3). Total dietary fibers and soluble fiber intakes were inversely linked with the ASV127 *Marseillibacter massiliensis* (*q* = 0.02, *q* = 0.08). Fat and animal protein intake were inversely associated with ASV26 *Bacteroides fragilis*, whereas carbohydrate intake was positively associated with it (*q* = 0.01). Strong negative relationships were found between ASV93 *Alistipes shahii* and several bile acids, including glycine-conjugated bile acids (i.e. GCA, GDCA, GCDCA) and taurine-conjugated bile acids (i.e. TDCA) (*q* < 0.05). Moreover, the baseline pro-inflammatory biomarkers CRP and TNF-α were inversely linked with ASV26 *Bacteroides fragilis* (*q* < 0.1), with IL-6 and TNF-α also inversely linked to ASV93 *Alistipes shahii* (*q* < 0.1). These data suggest that the recipient’s plant-based nutrient intake such as soluble fiber and lower levels of inflammation and blood lipids, and changes in bile acid concentrations may enhance the engraftment of the newly transferred microbiota.

To determine if the gut microbiota of the donor affected the engraftment results in the recipient, we ascertained the donors from whom the nine identified ASVs were effectively engrafted into the responders (Figure S4b). Figure S4a illustrates that both the responders and the non-responders received transplantation from one of four donors. There was no discernible distinction between non-responders and respondents regarding the distribution of donor composition. Moreover, the environmental fitting test analysis revealed that donor microbiota (PC1 of each donor microbiota) did not significantly associate with the variation of engrafted donors ASVs community between recipients (R^2^ = 0.069, *p* = .727, Figure 3c).

## Discussion

The use of FMT as a therapeutic strategy to modulate gut microbiota and host metabolism is currently limited by variable and transient engraftment and responses in recipients.^29^ There is still a lack of mechanistic understanding of the interactions between transferred donor microbiota and the existing host microbial community. In this study, a post hoc analysis of our previous clinical trial^22^ revealed that the baseline factors of recipients, including microbiota composition, diversity, diet, and microenvironmental characteristics were major determinants for donor microbial engraftment with improvement in insulin sensitivity as measured by HOMA2-IR.

Gut microbial diversity has been linked to human health, with lower diversity being associated with several acute and chronic diseases.^30,31^ Our findings demonstrated that the diversity of fecal microbiota was increased and strongly correlated with glucose homeostasis in participants undergoing FMT in combination with fiber treatment. Previous studies have revealed that following FMT for various conditions, recipients who show a clinical response generally experience an increase in gut microbiota diversity while those who do not show a clinical response often do not show an increase in microbial diversity.^32–36^ Furthermore, a large cross-sectional study in ~ 3400 individuals across the United States identified fasting insulin values to associate with microbiome diversity.^31^

In our study, participants with greater diversity at baseline experienced a smaller increase in diversity post-FMT with fiber supplementation, supporting the concept that microbial communities with greater diversity have enhanced resistance as well as resilience and are more resistant to change.^37,38^ While this resilience may be beneficial in healthy states, it may act as a barrier to the reversal of gut dysbiosis by FMT and to the implantation of new species,^39^ posing a challenge to the successful engraftment of FMT and minimizing potential metabolic benefits. However, while these results suggest that an increase in microbial diversity contributes to improved insulin sensitivity, these findings do not necessarily reflect causality.

In addition to diversity, the presence or absence of specific microbial taxa have also been linked to specific changes in host metabolic parameters.^8,31,40,41^ Responses to FMT have been linked to the taxonomic identity and strain abundance in both the donor and the recipient prior to FMT.^42^ In our analysis, ASVs engraftment revealed that responders had a significantly higher recipient-to-donor species colonization ratio than non-responders. Several donor-specific ASVs colonized successfully in the responders but not in non-responders including *Faecalibacillus intestinalis* (ASV44) and *Roseburia* spp. (ASV103), which are reported as species having glycosyl-transferases and dietary fiber degradation function, respectively,^43,44^ and which may play a role in improving insulin sensitivity through regulation of glucagon-like peptide-1^45,46^ and intestinal gluconeogenesis.^47^ Moreover, donor-specific *Christensenellaceae* spp. (ASV140), which was previously reported to be associated with metabolic health and reduced visceral adipose tissue and BMI,^25,48^ was identified in responders but absent in non-responders.

Host baseline nutrient intakes, including high intakes of sugar, animal fat and low intakes of fiber and thiamin, inflammatory profiles, blood cholesterol, LDL and bile acid levels were seen to be associated with the individualized impact of FMT on the improvement of microbial diversity and donor bacterial engraftment (i.e. ASV26 *Bacteroides fragilis*) in recipients. Diet has a significant role in shaping the gut microbiota by providing the microbes with substrates required for their proliferation and survival. A significant correlation was found between soluble fiber intakes at baseline and the engraftment community of donor ASVs in responders. Following FMT, the diet of the recipient may be crucial in providing for an environment to support the growth of the newly transplanted taxa. High fiber diets have been reported to impact gut microbiota composition and functional activity, largely through increasing available nutrients for microbial metabolism and growth. Thus, the relatively high initial fiber intakes may have provided a more conducive gut environment in the recipients that allowed for a higher donor ASVs engraftment during the FMT and fiber intervention. We also found a strong positive correlation between thiamin intake at baseline and improvement in microbial diversity. Thiamin, also known as Vitamin B1, is an essential coenzyme required for carbon metabolism,^49^ and its availability can influence microbial activity, interactions, and community structure.

In this study, we also found an inverse correlation between several bile acids including tauro-muricholic acid, glycocholic acid, glycodeoxycholic acid and glycochenodeoxycholic acid with α-diversity improvement and the engraftment of the newly transferred microbiota. Bile acids exert a potent antimicrobial effect by disrupting the bacterial cell wall architecture or by synthesizing the antimicrobial peptide cathelicidin, which influences the environment of gut microbiota by inhibiting the vegetative growth of bacteria.^50,51^ Secondary bile acids can also act as signaling molecules through both membrane and nuclear receptors, including G protein-coupled bile acid receptors (TGR5) and Farnesoid-X-receptor (FXR), to modulate cellular metabolism and insulin sensitivity.^52^ Together, our findings suggest that in addition to the baseline microbiota, diet, lipid metabolism, bile acids, and chronic inflammation may act as host environmental pressures that select for microbes with the necessary adaptive traits to colonize the gut long-term.

Our microbiome data sequenced bacterial 16S rRNA gene regions, limiting our analyzes to the response of fecal bacteria composition at higher resolution such as strain level. Although species identification in this study was based on ASV-level alignments with greater than 99% similarity, this should be carefully considered and further confirmed using metagenomics sequencing techniques. Future research should employ more comprehensive techniques, such as meta-omics^53^ and spatiotemporally resolved tools^54^ to analyze the bacterial dynamic information after transplantation at strain-level distinctions that likely drive individuality, as well as to achieve better functional profiles. Moreover, we acknowledge that the sample size of this exploration study was small thereby limiting the statistic power for conducting association among multi-datasets. Larger studies are required to develop robust machine learning algorithms and prediction models that identify the factors that predict clinical and microbiota responses to FMT and dietary fiber intervention.

## Conclusion

Our data demonstrate that the responders to FMT saw an increase in microbial diversity and increased donor-specific microbe engraftments, which were significantly associated with enhancement of insulin sensitivity. In addition, baseline recipient microbiota composition and related host factors, such as diet, inflammation, and bile acids, contributed to the restoration of the new microbial ecosystem. These findings further contribute to the advancement of the effect of microbiome-targeted therapies on metabolic syndrome by providing an ecological basis for the understanding of the individual variation in FMT efficiency. The findings of this secondary analysis may also serve as a foundation for enhancing the long-term restoration of the novel microbial community.

## Methods

### Registration

As previously described,^22^ this randomized controlled phase 2 proof-of-concept trial was registered in March 2018 at ClinicalTrials.gov (NCT03477916) to test the application of daily dietary fiber supplementation as an adjunct to FMT therapy to modify cardiometabolic outcomes in obese individuals with metabolic syndrome.

### Intervention

In brief, subjects with severe obesity (BMI: 45.3 ± 7.0 kg/m^2^) and metabolic syndrome were recruited from Edmonton’s Bariatric Clinic from 2018 to 2019 and randomized 1:1:1:1 into one of 4 groups: (1) Placebo FMT and cellulose (2) Placebo FMT and fermentable fiber; (3) FMT and cellulose; and (4) FMT and fermentable fiber. High-fermentable fiber supplementation consisted of an equal mixture by weight of soluble corn fiber (PROMITOR, Tate&Lyle, 114 kcal per 100 g), resistant wheat starch type 4 (Fibersym, MGP Ingredients, 35 kcal per 100 g), and acacia gum (Pre-Hydrated Gum Arabic, TIC GUMS, 17 kcal per 100 g). These fibers were selected due to their ability to promote beneficial gut microbial growth and immune modulation.^55–57^ Low-fermentable fiber supplementation consisted of microcrystalline cellulose (Microcel MC-12, Blanver Farmoquimica, 0 kcal per 100 g. Participants received a single dose of FMT (50 g donor stool) with 20 oral capsules followed by 6 weeks of daily fiber at a dose of 27 g/day (females) or 33 g/day (males). The inclusion criteria were described in the previous study.^22^ The dietary history questionnaire III was used to evaluate the dietary habits of study participants before treatment (DHQ3). The Diet History Questionnaire (DHQ) is an online food frequency questionnaire.^58^ DHQ3 consists of 135 food and beverage line items and 26 dietary supplement questions based on one month of intake.

#### Classifying of responders and non-responders

The primary outcome of the trial was the assessment of change in insulin sensitivity from baseline to 6 weeks using the HOMA2-IR. The total of 29 participants receiving FMT with fiber intervention were classified based on HOMA2-IR change from baseline to week 6 as responders (ΔHOMA-IR < 0, *n* = 21) and non-responders (ΔHOMA-IR > 0, *n* = 8).

### Blood metabolic markers, immunologic markers, and hormones analysis

After an overnight fast (>8 h), blood samples were collected and aliquots of plasma and serum were snap frozen in liquid nitrogen and stored at −80°C. An electrolyte panel, a liver panel, a glycemia panel, a lipid panel, and CRP were analyzed using standardized laboratory techniques as previously described.^22^ Change in insulin sensitivity between baseline and 6 weeks, as estimated by the HOMA-IR, was the primary outcome (HOMA2 Calculator, University of Oxford).

As previously described, IL-1β, IL-6, IL-10, TNF-α, and LPS were quantified by enzyme-linked immunosorbent assay (ELISA) as part of the systematic inflammation evaluation.^22^ Appetite and glucose metabolism-regulating hormones (leptin and ghrelin) were measured using a ghrelin assay (R&D Systems DuoSet ELISA, DY8149–05) and a leptin assay (R&D Systems DuoSet ELISA, DY398–05) (R&D Systems DuoSet ELISA, DY398–05).

### Plasma bile acids analysis

Targeted metabalomics analysis was performed for the quantification of bile acids in plasma using the AbsoluteIDQ bile acid kit (Biocrates, Inc) and liquid chromatography-mass spectrometry. This was performed at The Metabolomics Innovation Centre (Edmonton, Alberta, Canada) and includes quantification of unconjugated, taurine- and glycine-conjugated bile acids.

### Stool collection, short-chain fatty acid, and fecal microbiota analyses

Fecal samples were collected at baseline, 2 weeks, 6 weeks, and 12 weeks of treatment. Participants were provided with stool collection kits and instructed to collect samples within two days of their scheduled visits. The stool samples were preserved with at least 30 g of stool, stored at 4°C before transport, and delivered on ice. The samples were subsequently aliquoted and flash-frozen at −80°C after their arrival. The concentrations of stool SCFA were analyzed by gas chromatography using the previously described method.^59^

The DNA extraction method has been described in detail previously.^60^ Briefly, 0.1 g of stool was used to extract fecal DNA using the AquaStool protocol [MultiTarget Pharmaceuticals]. As per the manufacturer’s instructions, AquaRemove reagent [MultiTarget Pharmaceuticals] was added to further remove contaminating enzymatic inhibitors. The V4 region of the 16S rRNA gene was amplified by PCR with F515 and R806 primers, and Illumina sequencing was performed by the Genome Quebec Innovation Center [McGill University, Montreal, QC]. Using the Silva 132 pre-trained naïve Bayes classifier^61^ and the q2-feature-classifier plugin in the QIIME2 pipeline, taxonomic assignment (from kingdom to genus level) was performed on the representative sequences of each sample.^62^ A features table (amplicon sequence variants, ASVs) was constructed using DADA2.^63^ The sequence identity at species level was confirmed using representative sequences assignments of ASVs based on 16S rRNA gene databases on NCBI platforms (>99% similarity). QIIME2 was used to calculate the α-diversity using the Chao1 index. An even depth of 9518 sequences per sample was used to conduct microbiome diversity and composition analyzes. The rarefaction curve of all samples is shown in Figure S5.

### Statistical Analysis

R v4.1.1 and GraphPad Prism v9.5.2 were used to conduct statistical analyzes. To assess the degree of association between microbial engraftment (changes in α-diversity and donor-specific ASVs) and host baseline factors (anthropometric measurements, metabolic markers, immune cytokines, bile acids, and macronutrient intakes), repeated measures linear mixed model regression with donor selection adjustment was used. ANCOVA, with donor selection as a covariable, was used to evaluate the differences between groups. Normality was assessed by visual inspection of residual and histogram plots.

In terms of longitudinal analyzes on the shifts of donor-specific ASVs colonization, the main effect of time and group were assessed using linear mixed model regression. At each time point, the differences between groups (responders vs. non-responders) were evaluated using a t-test with Welch correction. We applied wcmdscale function in Vegan package (version 2.5–7)^64^ to conduct MDS ordination based on the Bray-Curtis dissimilarity matrix of the engrafted donor-specific ASVs composition between FMT patients at week 6. The envfit function was used to assess which baseline factors were key features explaining the donor-specific ASVs community variation.

To identify potential determinants that predicted the effects of FMT and fiber supplementation on HOMA-IR response at week 6, separate RFCs were independently trained on changes in donor microbial composition, recipient baseline microbiome-related factors (microbial composition, bile acids, immunometabolic biomarkers, and dietary intakes). Random Forest employs supervised tree-based machine learning algorithms that are supposedly robust for the discriminant analysis of high-dimensional, small sample-size data.^65–67^ Prior to analysis, participants were categorized as responders or non-responders (decreased vs. increased HOMA2-IR). Independent RFCs were performed using the default settings of the random Forest package.^68^ The generalization error of each RFC was estimated across 100 replicates using leave-one-out cross-validation as previously described.^69^ To evaluate the performance of each RFC, AUC-ROCs were generated from the true possible cross-validated results using the pROC package.^70^ RFCs with AUC-ROC values ≥ 0.7 and out-of-bag error rates < 0.6 were considered to have good prediction accuracy.^67^ To determine the importance of each variable in classifying responders vs. non-responders, the average mean importance scores of 100 replicates were calculated. To support the RFCs as having a direction of the association, a *t*-test was conducted to compare the difference in the levels of the best predictors between respondents and non-responders.

## Supplementary Material

Supplemental Material

## Data Availability

The raw sequencing data have been deposited into the Sequence Read Archive (SRA) of the NCBI (http://www.ncbi.nlm.nih.gov/sra) under BioProject PRJNA708262. All other relevant data related to the current study are freely available from the corresponding author (K.L.M.) upon request, which does not include confidential patient information.
