# Supplementary figures and images for: Recipient microbiome-related features predicting metabolic improvement following fecal microbiota transplantation in adults with severe obesity and metabolic syndrome: a secondary analysis of a phase 2 clinical trial
Source: Gut Microbes. 2024 Apr 29;16(1):2345134. doi: 10.1080/19490976.2024.2345134 (PMC11062372; doi:10.1080/19490976.2024.2345134)

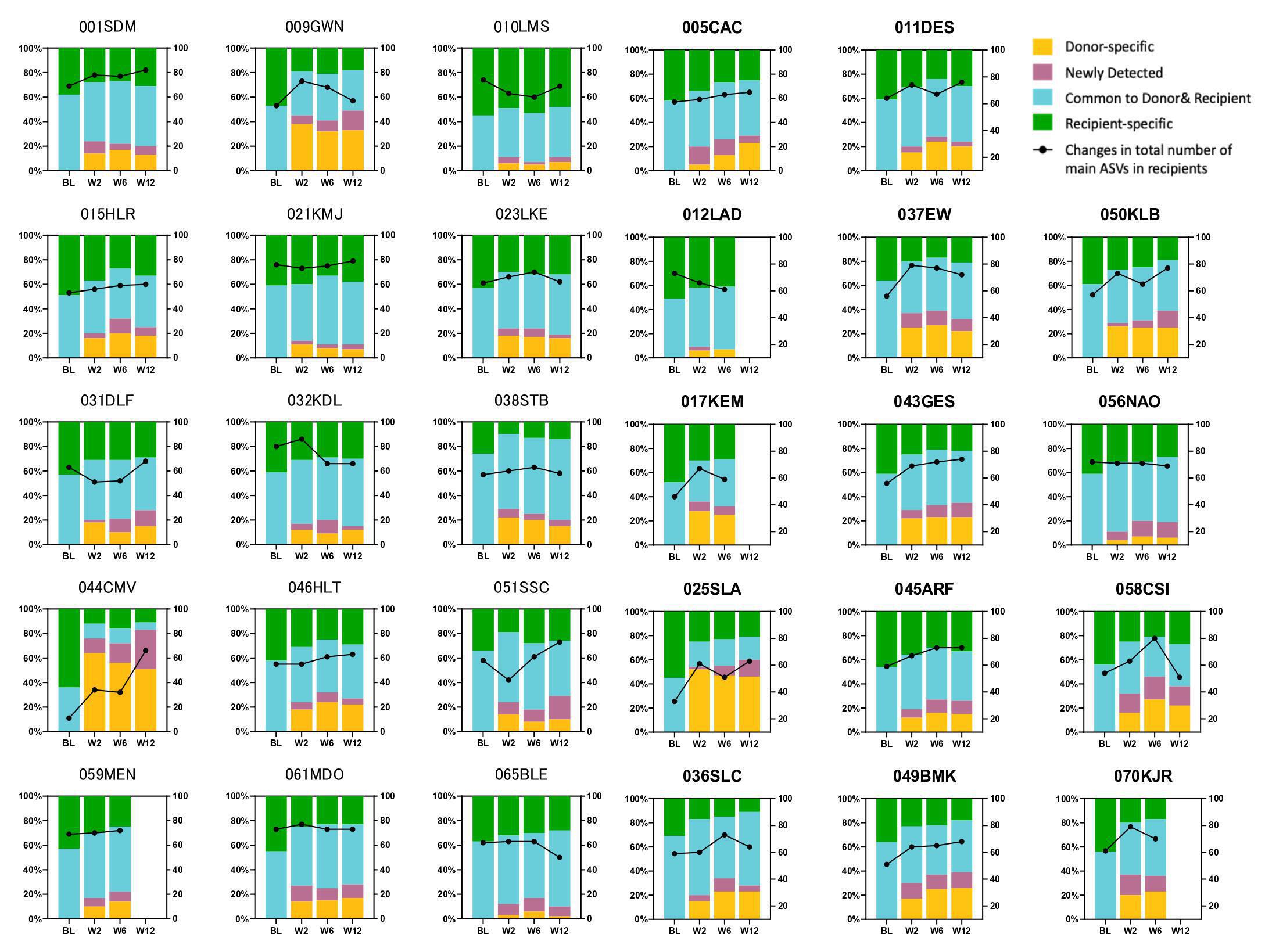

Supplement: Supplemental Material [file KGMI_A_2345134_SM4028.zip › Fig S1.jpg]

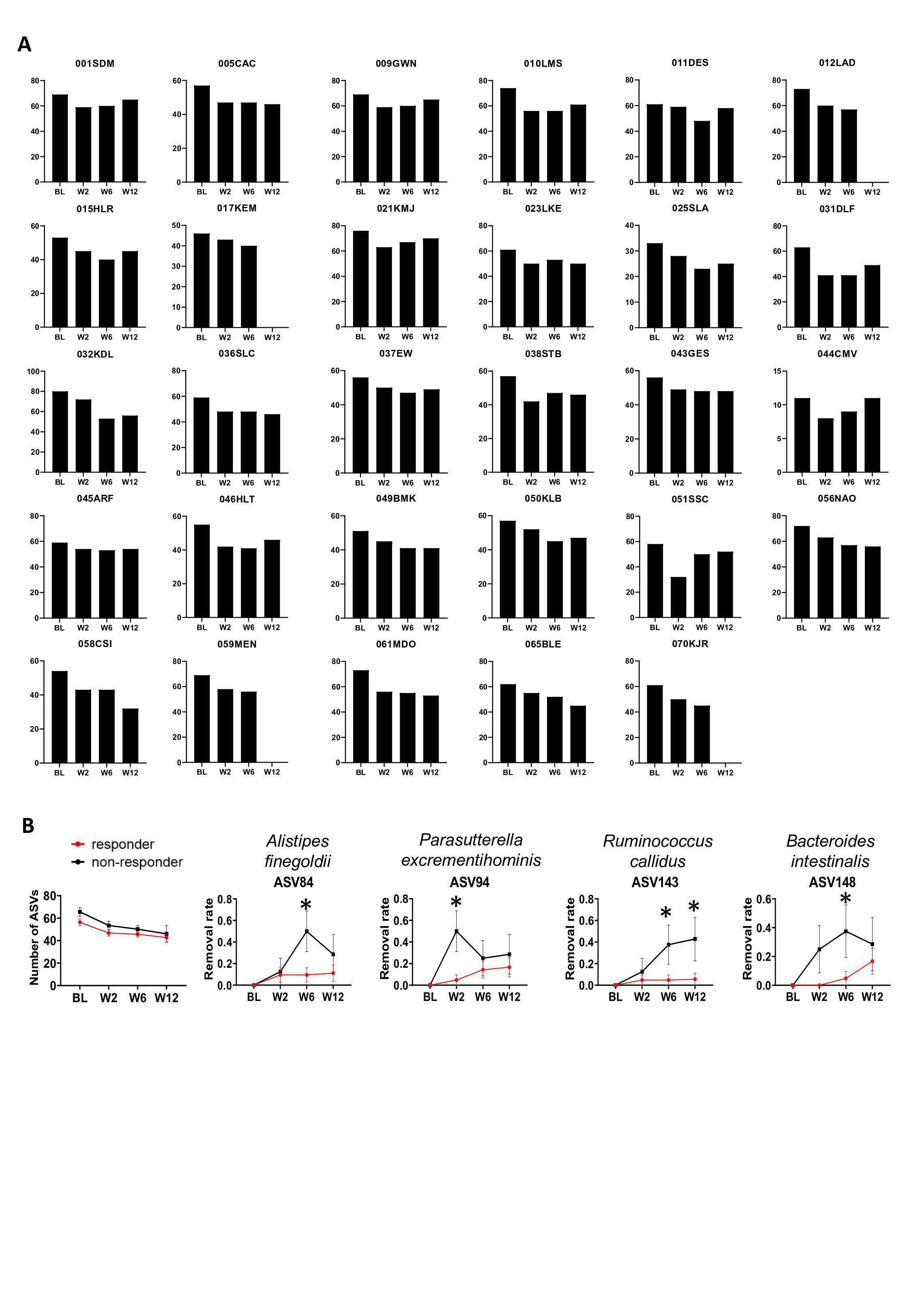

Supplement: Supplemental Material [file KGMI_A_2345134_SM4028.zip › Fig S2.jpg]

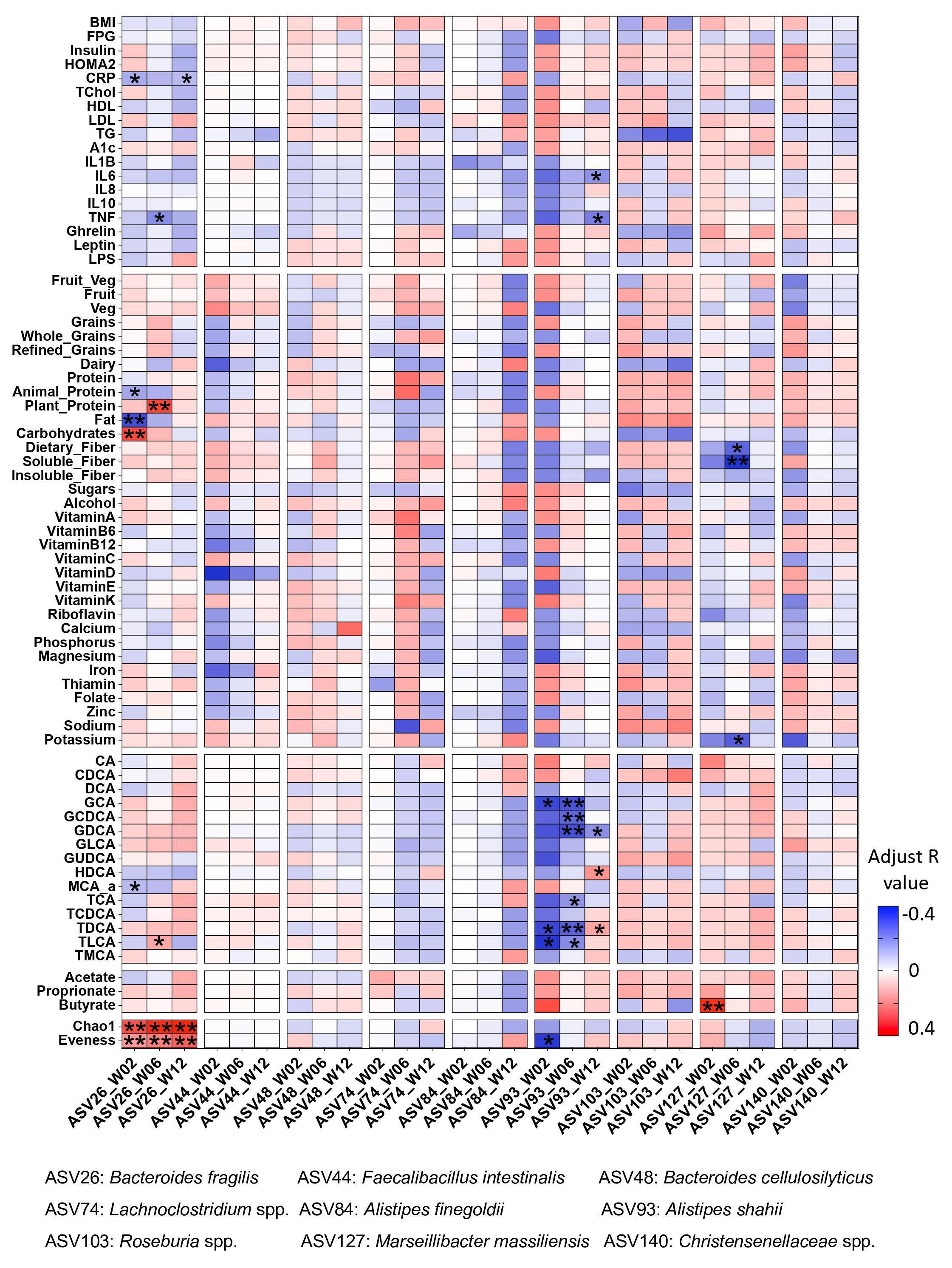

Supplement: Supplemental Material [file KGMI_A_2345134_SM4028.zip › Fig S3.jpg]

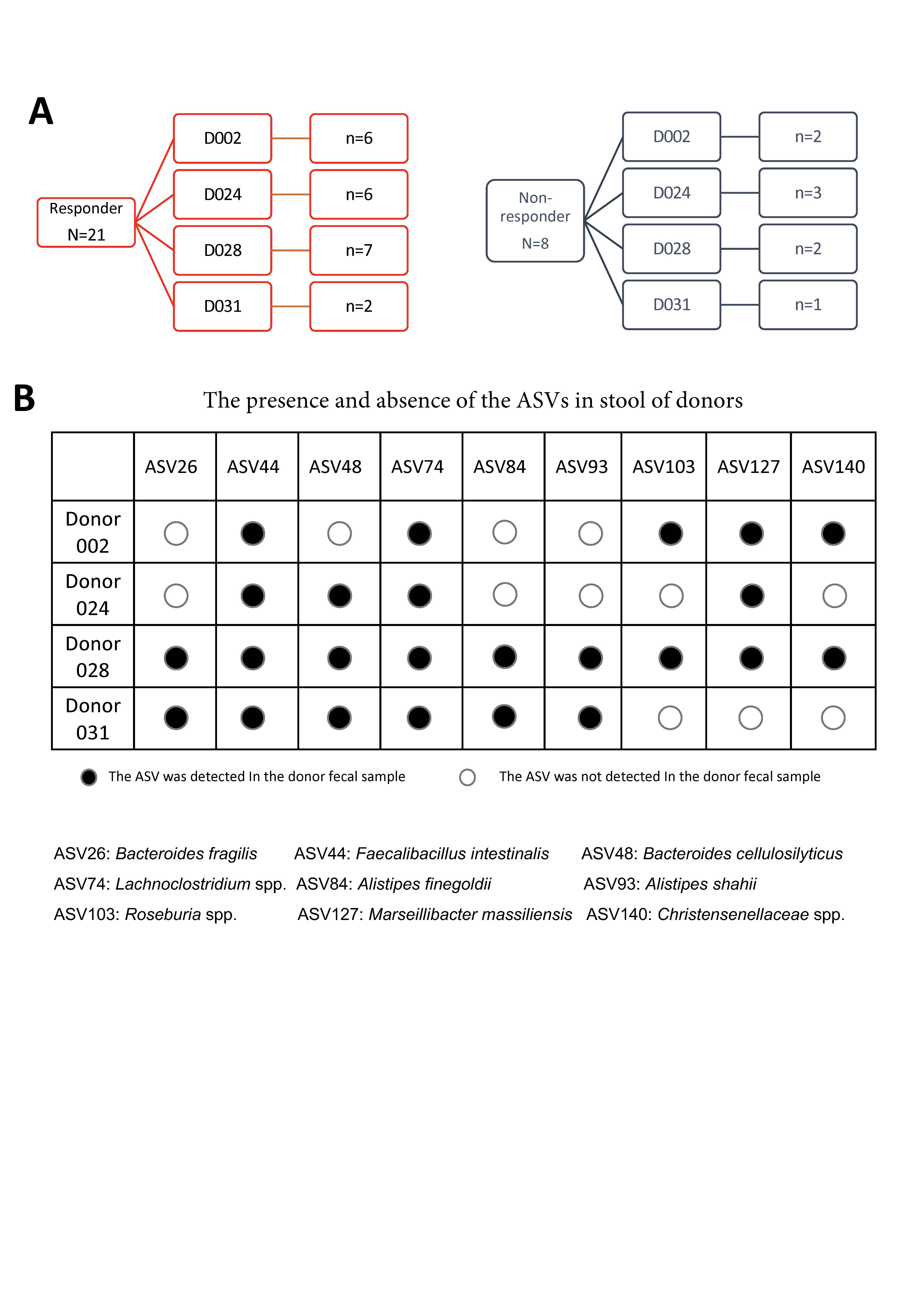

Supplement: Supplemental Material [file KGMI_A_2345134_SM4028.zip › Fig S4.jpg]

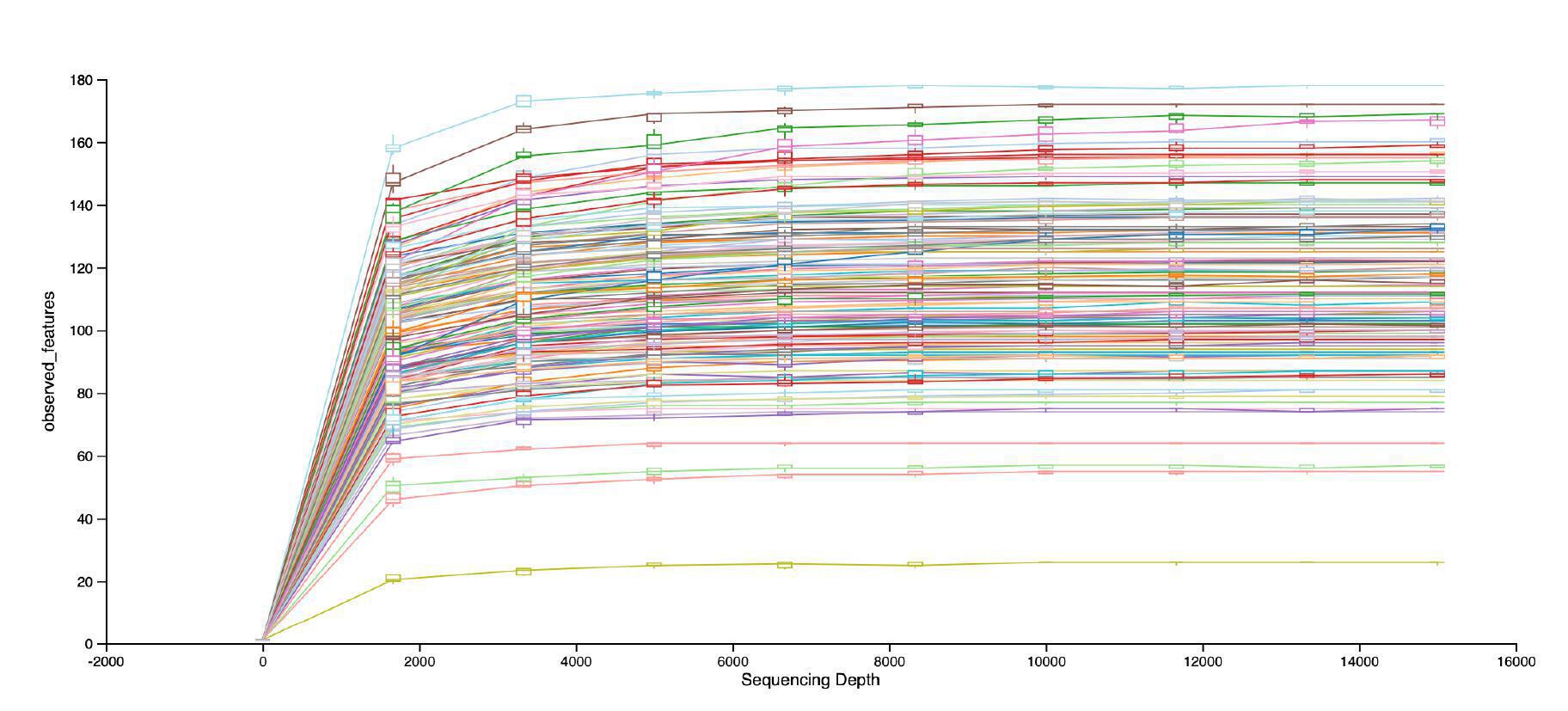

Supplement: Supplemental Material [file KGMI_A_2345134_SM4028.zip › Fig S5.jpg]
